# Double Jeopardy: Risks of Opioid–Benzodiazepine Co-prescription in Older Adults with Gastrointestinal Cancer

**DOI:** 10.1245/s10434-026-19233-9

**Published:** 2026-02-19

**Authors:** Areesh Mevawalla, Azza Sarfraz, Jasmine King, Qaidar Alizai, Meher Angez, Odysseas P. Chatzipanagiotou, Mujtaba Khalil, Timothy M. Pawlik

**Affiliations:** 1 Department of Surgery, The Ohio State University Wexner Medical Center and James Comprehensive Cancer Center, Columbus, OH USA; 2 Department of Surgery, University of Wisconsin-Madison, Madison, WI USA

**Keywords:** Gastrointestinal cancer, Opioids, Benzodiazepines, Co-prescription, Older adults, Overdose, Falls, Hospitalization, Mortality, SEER–Medicare

## Abstract

**Background:**

Opioid analgesics and benzodiazepines are frequently prescribed to manage pain, anxiety, and insomnia in older adults with gastrointestinal (GI) cancers, yet outcomes of concurrent use are incompletely defined.

**Patients and Methods:**

Using linked Surveillance, Epidemiology, and End Results (SEER)–Medicare (2014–2018), daily exposure was classified as no opioid/benzodiazepine, opioid only, or opioid–benzodiazepine co-prescription and overdose; falls/fractures, all-cause hospitalization, and all-cause mortality within 12 months were assessed. Time-varying Cox models compared opioid-only and co-prescription versus no exposure, and for incremental risk, co-prescription versus opioid-only.

**Results:**

Among 26,896 patients (median age 74 years; 53.1% female), 5086 (18.9%) received concurrent opioid–benzodiazepine prescriptions and 2288 (8.5%) received opioids alone. Within 12 months, among patients unexposed, opioid-only, and co-prescribed, unadjusted first-event rates per 100,000 person-days (95% CI) were: falls/fractures 27.8 (26.7–28.9), 55.0 (47.1–63.9), and 61.0 (50.8–72.5); all-cause hospitalizations 393.0 (388.0–398.0), 439.0 (410.0–470.0), and 441.0 (406.0–480.0); overdose 1.40 (1.20–1.70), 6.20 (3.80–9.50), and 8.80 (5.32–13.83); and mortality 11.5 (10.8–12.2), 20.6 (15.9–26.1), and 29.6 (22.8–37.9), respectively (global *p* < 0.001). In adjusted models versus no exposure, both opioid-only and co-prescribed patients had higher hazards of adverse outcomes. Using opioid-only as reference, co-prescription was associated with incremental hazards of all-cause hospitalization (HR 1.12, 95% CI 1.03–1.23) and all-cause mortality (HR 1.35, 95% CI 1.06–1.73).

**Conclusions:**

Opioid exposure was associated with elevated risk, while the addition of benzodiazepines conferred incremental increases in hospitalization and mortality versus opioid-only use.

**Supplementary Information:**

The online version contains supplementary material available at 10.1245/s10434-026-19233-9.

Gastrointestinal (GI) malignancies, including colorectal, pancreatic, biliary tract, and primary liver cancers, impose a substantial health burden worldwide, accounting for roughly one-quarter of new cancer cases and one-third of cancer-related deaths globally.^1,2^ In the USA, these tumors represent a significant share of cancer mortality; colorectal and pancreatic cancer alone each claim roughly 50,000 lives annually.^3,4^ Patients with advanced GI cancers often experience profound symptom burdens, notably severe pain, psychological distress (e.g., anxiety), and insomnia.^5^ Effective management of these symptoms frequently requires pharmacotherapy, with opioid analgesics remaining the cornerstone of cancer pain control and benzodiazepines widely prescribed for anxiety and sleep disturbances.^6^

Opioids and benzodiazepines are therefore commonly co‐utilized in oncology care, despite guidelines urging caution with concurrent use.^7^ Prior studies show that opioid–benzodiazepine co-prescribing is frequent; one U.S. analysis reported that the proportion of opioid users also receiving benzodiazepines nearly doubled from 9 to 17% between 2001 and 2013, and that such co-use is linked to adverse outcomes.^8^ Concomitant use of these drugs exerts synergistic depressant effects on the central nervous system, contributing to higher risks of adverse events.^8,9^ These safety concerns are particularly salient in older adults, who are more vulnerable to sedation and falls.^10^ However, recent oncology-specific evidence suggests that co-prescribing may not uniformly confer incremental risk beyond opioid use alone; for example, Check et al. reported limited additional risk attributable to concurrent benzodiazepine use compared directly with opioid-only exposure in an older cancer population.^6^ Notably, while several investigations have examined opioid and benzodiazepine co-use in broad cancer or general populations, few have focused on patients with GI cancer malignancies or explored how risks vary by treatment duration, dosage, or patient characteristics.^6,9^ This gap in literature limits understanding of how best to balance symptom relief and safety in this high-risk group.

Recognizing the paucity of evidence specific to GI malignancies, and noting that prior work in broader oncology populations has reported mixed findings regarding incremental risk from co-prescription beyond opioid use alone, including analyses suggesting limited or no additional risk for certain outcomes, we designed a national cohort study assessing the association between opioid–benzodiazepine co-prescribing and key clinical outcomes in older adults with colorectal, pancreatic, biliary, or liver cancers.^6^ Using linked Surveillance, Epidemiology, and End Results (SEER)–Medicare data, we applied time-varying exposure analyses to evaluate whether concurrent opioid and benzodiazepine use is associated with increased risks of opioid overdose, falls/fractures, hospitalizations, and all-cause mortality. Findings from this study will inform safer pain and symptom management strategies in GI oncology, guiding clinicians in optimizing analgesia and anxiolysis while minimizing harm in vulnerable older patients.

## Patients and Methods

### Data Source, Study Population, and Cohort Selection

Linked Surveillance, Epidemiology, and End Results–Medicare (SEER–Medicare) files were used to identify older adults with GI cancers and to ascertain prescription exposures and outcomes.^11^ The analytic cohort included individuals aged 66–99 years diagnosed with a first primary malignancy of the liver, intrahepatic or extrahepatic bile ducts, gallbladder, pancreas, colon and rectum diagnosed between 1 January 2014, and 31 December 2018, identified via SEER site recodes and ICD-O-3 topography codes.^12^ Continuous enrollment in Medicare Parts A and B with no health maintenance organization participation was required for the 12 months preceding diagnosis and the 12 months following diagnosis (or until death). Continuous Part D enrollment was required from 3 months before diagnosis through 12 months after diagnosis (or until death). Patients diagnosed by death certificate or autopsy only, those with in situ disease, secondary cancers, implausible temporal ordering of diagnosis and treatment dates, or lacking a valid county FIPS code were excluded. The Institutional Review Board at The Ohio State University waived informed consent because the data were analyzed under a SEER–Medicare Data Use Agreement.

### Exposure and Covariates

The primary exposure was daily concurrent use of opioids and benzodiazepines defined from the Medicare Part D Prescription Drug Event (PDE) file. Dispensing date and days’ supply were used to construct a day-level indicator of possession. On each day, beneficiaries were classified into three mutually exclusive, time-varying categories: no opioid or benzodiazepine; opioid only; or opioid + benzodiazepine (co-prescribed). Benzodiazepine-only exposure was rare (*n* ≈ 1) and was therefore not modeled as a separate group. Opioids were identified using National Drug Codes and converted to morphine milligram equivalents (MME); buprenorphine formulations were excluded.^13^ Benzodiazepines were classified by generic name. High-dose opioid exposure was defined as ≥ 50 MME/day.^14^ Continuous co-prescribing was defined as ≥ 90 consecutive overlap days of opioid and benzodiazepine possession; shorter overlap was classified as intermittent.^15^

Covariates included age, sex, race/ethnicity, U.S. Census region, metropolitan status, social vulnerability index (SVI), and tumor characteristics including primary GI site, SEER Summary Stage (local/regional versus distant; unknown retained). Comorbidity burden was measured using the Charlson comorbidity index (CCI) on the basis of inpatient, outpatient, and carrier claims in the year prior to diagnosis.^16^ Frailty was assessed using a claims-based frailty index constructed from diagnosis and utilization codes in the year preceding cancer diagnosis, capturing domains of functional impairment, mobility limitation, cognitive dysfunction, and health service dependence, consistent with previously validated approaches in SEER–Medicare populations.^17^ Mental health and substance use disorders (e.g., depression and anxiety) were identified from chronic conditions warehouse indicators and corroborated with psychotropic medication fills.^11^ Time-varying indicators for surgery, chemotherapy, and radiation were identified using ICD-9-CM/ICD-10-PCS, CPT, and HCPCS codes. Missing covariate data < 10% were handled by complete-case analysis; variables with higher missingness included an “unknown” category.

### Outcomes of Interest

Four primary outcomes were examined within 12 months of diagnosis: opioid overdose, falls/fractures, all-cause hospitalization, and all-cause mortality. Overdoses were identified from inpatient and emergency claims using validated ICD-9-CM/ICD-10-CM algorithms, with sensitivity definitions including related respiratory failure codes.^18^ Falls and fractures were identified using Agency for Healthcare Research and Quality and CMS claims algorithms.^19^ Hospitalizations were ascertained from MedPAR files, with recurrent admissions retained for counting-process analyses. Mortality data were obtained from SEER and the Master Beneficiary Summary File. Outcomes were coded on a daily basis to align with exposure classification.

### Statistical Analysis

Baseline characteristics were summarized as medians with interquartile ranges for continuous variables and counts with percentages for categorical variables and compared across exposure categories using the Kruskal–Wallis and *χ*^2^ tests, respectively. Unadjusted event rates per 100,000 person-days were calculated as the number of first events divided by total person-time at risk, and 95% confidence intervals and global *p*-values for unadjusted comparisons were estimated using Poisson regression models with a log (person-time) offset and robust standard errors. The association between time-varying exposure and each endpoint were estimated using Cox proportional hazards models adjusted for demographics, tumor characteristics, comorbidity, mental health/substance use, SVI tertile, frailty, dual eligibility, region, year, and time-varying treatment indicators, using no exposure as the primary reference group; parallel models using opioid-only exposure as the reference were fit to estimate incremental risk attributable to benzodiazepines among opioid-treated patients. Treatment indicators were coded as time-varying (switching from 0 to 1 on the procedure/first treatment date and remaining 1 thereafter) to ensure temporal ordering and avoid immortal time or reverse-causation bias. Robust standard errors were clustered at the beneficiary level. Recurrent hospitalizations were modeled using Andersen–Gill extensions with robust variance. Site fixed effects were included to account for heterogeneity across cancer types; SEER registry fixed effects were included in all models. Additional analyses evaluated heterogeneity by high-dose opioid exposure (≥ 50 MME/day), and continuous versus intermittent co-prescribing. Predictors of any co-prescribing were examined using multivariable logistic regression with robust standard errors and SEER registry fixed effects. Two-sided *p*-values < 0.05 were considered statistically significant. Analyses were performed using Stata 18 (StataCorp, College Station, TX).

## Results

### Baseline Characteristics

Among 26,896 older adults with GI cancers—11,190 colon (41.6%), 7471 pancreatic (27.8%), 3403 rectal (12.7%), 2784 hepatic (10.4%), and 1275 biliary tract (4.7%)—19,521 (72.6%) received no opioids or benzodiazepines, 2288 (8.5%) received opioids alone, and 5086 (18.9%) were co-prescribed. Compared with unexposed patients, those who were co-prescribed were younger (median 73 versus 74 years), more often female (*n* = 3008, 59.1% versus *n* = 9946, 50.1%), and white (*n* = 4165, 81.9% versus *n* = 14,381, 73.7%) (all *p* < 0.001). Co-prescribed patients also had a higher comorbidity burden (CCI > 2: *n* = 833, 16.4% versus *n* = 2813, 14.4%), were more likely to reside in high-SVI counties (*n* = 1850, 36.4% versus *n* = 6724, 34.5%), and had greater prevalence of anxiety (*n* = 423, 8.3% versus *n* = 526, 2.7%) (all *p* < 0.001). By cancer site, pancreatic cancer was more common among patients with co-prescription (*n* = 1910, 37.6% versus *n* = 4938, 25.3%), while rectal cancer was less common (n = 597, 11.7% versus *n* = 2470, 12.7%). Stage IV disease occurred more frequently in the co-prescribed group (*n* = 1956, 38.5% versus *n* = 5704, 29.2%). Of note, patients with co-prescription were less likely to undergo cancer-directed surgery within 1 year of diagnosis (*n* = 2387, 46.9% versus *n* = 11,217, 57.5%), had higher receipt of chemotherapy [co-prescribed: 4180 (82.2%) versus unexposed: 14,038 (71.9%)] and radiation [co-prescribed: 1164 (22.9%) versus unexposed: 3423 (17.5%)] (all p < 0.001) (Table 1).

**Table 1:** Baseline characteristics of patients with gastrointestinal cancer

Characteristics	Total (*n* = 26,896)	No opioids or benzodiazepines (*n* = 19,521, 72.6%)	Opioids only (*n* = 2288, 8.5%)	Opioids and benzodiazepines (*n* = 5086, 18.9%)	*p*-Value
Age, years, median [IQR]	74 (74–75)	74 (74–75)	74 (73–74)	73 (73–73)	0.02
Sex					< 0.001
Male	12,613 (46.9)	9575 (49.1)	959 (41.9)	2078 (40.9)	
Female	14,283 (53.1)	9946 (50.1)	1329 (58.1)	3008 (59.1)	
Race					< 0.001
White	20,379 (75.8)	14,381 (73.7)	1832 (80.1)	4165 (81.9)	
Black	2188 (8.1)	1782 (9.1)	104 (4.6)	302 (5.9)	
Hispanic	2266 (8.4)	1700 (8.7)	190 (8.3)	376 (7.4)	
Other	2063 (7.7)	1658 (8.5)	162 (7.1)	243 (4.8)	
Area					< 0.001
Metropolitan	22,187 (82.5)	16,019 (82.1)	1948 (85.1)	4219 (83.0)	
Non-metropolitan	4709 (17.5)	3502 (17.9)	340 (14.9)	867 (17.1)	
Region					< 0.001
Midwest	2164 (8.1)	1576 (8.10)	165 (7.2)	423 (8.3)	
Northeast	1872 (7.0)	1366 (7.0)	121 (5.3)	385 (7.6)	
South	9606 (35.7)	7127 (36.5)	782 (34.2)	1697 (33.4)	
West	13,254 (49.3)	9452 (48.4)	1220 (53.3)	2581 (50.8)	
CCI					< 0.001
≤ 2	22,995 (85.5)	16,708 (85.6)	2033 (88.9)	4253 (83.6)	
> 2	3901 (14.5)	2813 (14.4)	255 (11.2)	833 (16.4)	
SVI					< 0.001
Low	8804 (32.8)	6335 (32.5)	873 (38.2)	1596 (31.4)	
Moderate	8831 (32.9)	6453 (33.1)	740 (32.4)	1638 (32.2)	
High	9249 (34.4)	6724 (34.5)	674 (29.5)	1850 (36.4)	
Anxiety					< 0.001
Yes	1062 (3.9)	526 (2.70)	113 (4.9)	423 (8.3)	
Depression					0.14
Yes	1055 (3.9)	772 (4.0)	70 (3.1)	213 (4.2)	
Frailty score					
First quartile (Lowest)	7050 (26.2%)	5650 (29.0%)	520 (22.7%)	880 (17.3%)	< 0.001
Second quartile	6950 (25.8%)	5150 (26.4%)	600 (26.2%)	1200 (23.6%)	
Third quartile	6700 (24.9%)	4650 (23.8%)	610 (26.7%)	1440 (28.3%)	
Fourth quartile (Highest)	6196 (23.1%)	4071 (20.9%)	558 (24.4%)	1567 (30.8%)	
Cancer site					
Colon	11,190 (41.6)	8496 (43.5)	991 (43.3)	1703 (33.5)	< 0.001
Rectum	3403 (12.7)	2470 (12.7)	336 (14.7)	597 (11.7)	
Biliary tract	1275 (4.7)	854 (4.5)	108 (4.7)	312 (6.1)	
PDAC	7471 (27.8)	4938 (25.3)	623 (27.2)	1910 (37.6)	
Liver	2784 (10.4)	2188 (11.2)	164 (7.2)	423 (8.5)	
Stage					< 0.001
1	5890 (21.9)	4561 (23.4)	440 (19.2)	889 (17.5)	
2	6828 (25.4)	5035 (25.8)	615 (26.9)	1178 (23.2)	
3	5898 (21.9)	4221 (21.6)	614 (26.8)	1063 (20.9)	
4	8280 (30.8)	5704 (29.2)	619 (27.1)	1956 (38.5)	
Surgical therapy					< 0.001
Yes	15,075 (56.1)	11,217 (57.5)	1471 (64.3)	2387 (46.9)	
Chemotherapy					< 0.001
Yes	20,167 (75.0)	14,038(71.9)	1948 (85.1)	4180 (82.2)	
Radiation therapy					< 0.001
Yes	5164 (19.2)	3423(17.5)	577 (25.2)	1164 (22.9)	

1 Values are reported as *n* (column %) unless otherwise indicated. Percentages may not sum to 100 due to rounding

2 Age is presented as median (interquartile range [IQR])

3 Cancer site classification was based on SEER site recodes and ICD-O-3 topography codes (see Supplementary Table 2)

4 Stage reflects SEER Summary Stage at diagnosis

5 Charlson comorbidity index (CCI) was calculated from inpatient, outpatient, and carrier claims in the year prior to diagnosis

6 Social vulnerability index (SVI) tertiles were assigned using CDC/ATSDR county-level data linked by FIPS code

7 Anxiety and depression were identified using chronic conditions warehouse (CCW) indicators and corroborated with psychotropic medication fills

8 *p*-Values are from *χ*^2^ tests for categorical variables and Kruskal–Wallis tests for continuous variables

### Prescription Patterns and Outcome Risks

Within 1 year of diagnosis, unadjusted first-event rates [per 100,000 person-days (95% CI)] differed significantly across exposure groups. Overdose occurred at 8.8 (5.3–13.8), 6.2 (3.8–9.5), and 1.4 (1.2–1.7) per 100,000 person-days in the co-prescribed, opioid-only, and unexposed groups, respectively. Rates of falls or fractures were 61.0 (50.8–72.5), 55.0 (47.1–63.9), and 27.8 (26.7–28.9); hospitalizations 441.0 (406.0–480.0), 439.0 (410.0–470.0), and 393.0 (388.0–398.0); and mortality 29.6 (22.8–37.9), 20.6 (15.9–26.1), and 11.5 (10.8–12.2), respectively. Global *p*-values from Poisson regression models with a log (person-time) offset were all < 0.001. (Table 2).

**Table 2:** Unadjusted first-event and rates of adverse outcomes within 12 months after cancer diagnosis, per 100,000 person-days

	No opioids or benzodiazepines	Only opioid	Opioid and benzodiazepines	All patients	*p*-Value*
Falls/fracture	27.8 (26.7–28.9)	55.0 (47.1–63.9)	61.0 (50.8–72.5)	76.3 (74.6–78.1)	< 0.001
All-cause hospitalizations	393.0 (388.0–398.0)	439.0 (410.0–470.0)	441.0 (406.0–480.0)	600.6 (595.7–605.4)	< 0.001
Opioid overdose	1.40 (1.20–1.70)	6.20 (3.80–9.50)	8.80 (5.32–13.83)	3.30 (3.01–3.72)	< 0.001
All-cause mortality	11.5 (10.8–12.2)	20.6 (15.9–26.1)	29.6 (22.8–37.9)	12.2 (11.5–12.9)	< 0.001

Event rates calculated as the number of first events divided by total person-days at risk, multiplied by 100,000. Person-days were accrued from diagnosis until first event, death, or censoring at 12 months. *p-Values derived from Poisson regression models comparing exposure groups with log(person-time) offset and robust standard errors. Rates are per 100,000 person-days: 95 % confidence intervals in parentheses

In multivariable Cox regression models for first events, both opioid-only and co-prescribed patients had elevated risks relative to unexposed patients (Table 3). Overdose risk was markedly increased, with a more than fourfold higher hazard among co-prescribed patients (HR 4.60, 95% CI 2.75–7.70) and approximately threefold higher hazard among opioid-only users (HR 3.05, 95% CI 1.85–5.00). Risks of falls and fractures were also increased (HR 1.95, 95% CI 1.65–2.30 for co-prescription; HR 1.78, 95% CI 1.50–2.10 for opioid-only), and mortality followed a similar pattern (HR 1.85, 95% CI 1.45–2.35 versus HR 1.32, 95% CI 1.05–1.66). Hospitalization risk was modestly elevated with co-prescription (HR 1.13, 95% CI 1.05–1.23) but not with opioid-only use (HR 1.01, 95% CI 0.90–1.08) (Table 3). Findings were consistent in Andersen–Gill models accounting for recurrent events (Table 3). Compared with unexposed patients, overdose risk remained elevated among those co-prescribed (HR 4.75, 95% CI 2.85–7.90) and among opioid-only users (HR 3.15, 95% CI 1.90–5.20). Risks of falls and fractures were also elevated (HR 1.98, 95% CI 1.66–2.36 for co-prescription; HR 1.72, 95% CI 1.47–2.01 for opioid-only). Hospitalization risk was modestly increased with co-prescription (HR 1.08, 95% CI 1.01–1.17) but not with opioid-only use (HR 0.95, 95% CI 0.90–1.01) (Table 3).

**Table 3:** Adjusted hazard ratios (HRs) for adverse events within 12 months after cancer diagnosis, by opioid and benzodiazepine exposure*

	Model type	No opioids or benzodiazepines	Opioid only	Opioids and benzodiazepines	Incremental HR opioid and benzodiazepines (ref. opioid only)
			HR (95% CI)	HR (95% CI)	HR (95% CI)
Falls/fractures	First event Cox	Ref.	1.78 (1.50–2.10)	1.95 (1.65–2.30)	1.10 (0.88–1.36)
	Andersen–Gill^†^	Ref.	1.72 (1.47–2.01)	1.98 (1.66–2.36)	1.15 (0.91–1.45)
All-cause hospitalizations	First event Cox	Ref.	1.01 (0.90–1.08)	1.13 (1.05–1.23)	1.12 (1.03–1.23)
	Andersen–Gill^†^	Ref.	0.95 (0.90–1.01)	1.08 (1.01–1.17)	1.13 (1.05–1.22)
Overdose	First event Cox	Ref.	3.05 (1.85–5.00)	4.60 (2.75–7.70)	1.45 (0.72–2.95)
	Andersen–Gill^†^	Ref.	3.15 (1.90–5.20)	4.75 (2.85–7.90)	1.48 (0.74–3.00)
All-cause mortality	First event Cox	Ref.	1.32 (1.05–1.66)	1.85 (1.45–2.35)	1.35 (1.06–1.73)

*All models adjusted for age group, sex, race/ethnicity, cancer site, stage, comorbidity (Charlson), frailty, and time-varying cancer treatments (surgery, chemotherapy, radiation)

†Andersen–Gill models account for recurrent events (falls, hospitalizations, overdose); HRs reflect the relative hazard of each event, not just the first occurrence

To directly assess incremental risk attributable to co-prescription use, models were refit using opioid-only exposure as the reference group. In multivariable Cox regression models for first events, co-prescribed patients demonstrated higher hazards of hospitalization and mortality relative to those receiving opioids alone (HR 1.12, 95% CI 1.03–1.23 and HR 1.35, 95% CI 1.06–1.73, respectively). The relative hazards for overdose (HR 1.45, 95% CI 0.72–2.95) and falls or fractures (HR 1.10, 95% CI 0.88–1.36) were numerically higher but not statistically significant. Findings were consistent in Andersen–Gill models accounting for recurrent events, in which co-prescription remained associated with increased hospitalization risk compared with opioid-only use (HR 1.13, 95% CI 1.05–1.22), while incremental associations with overdose (HR 1.48, 95% CI 0.74–3.00) and falls or fractures (HR 1.15, 95% CI 0.91–1.45) were not statistically significant (Table 3).

### Comparative Risks of Continuous and Intermittent Co-prescription

**Fig. 1:**
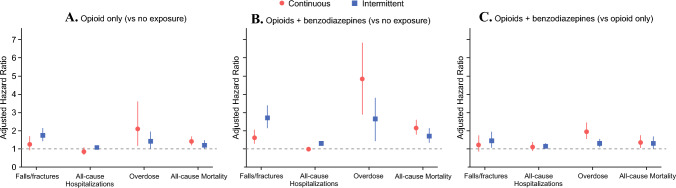
Adjusted risks of adverse outcomes by intermittent versus continuous exposure patterns; adjusted hazard ratios (HRs) with 95% confidence intervals for falls/fractures, all-cause hospitalizations, overdose, and all-cause mortality among older adults with gastrointestinal cancers, stratified by exposure pattern (continuous versus intermittent); panel **A** displays opioid-only exposure versus no opioid/no benzodiazepine (reference), panel **B** displays opioid–benzodiazepine co-prescription versus no opioid/no benzodiazepine (reference), and panel **C** displays the incremental association of co-prescription versus opioid-only exposure (reference), shown separately for continuous and intermittent use; models adjusted for demographics, tumor characteristics, comorbidities, mental health and cancer-directed treatment, frailty and county-level factors

In adjusted Cox models, relative to those unexposed, intermittent co-prescription was more strongly associated with falls and fractures (HR 2.70, 95% CI 2.15–3.40 versus HR 1.62, 95% CI 1.28–2.05 for continuous use) and with all-cause hospitalizations (HR 1.30, 95% CI 1.18–1.44 versus HR 0.98, 95% CI 0.87–1.10 for continuous use) (Table S1). In contrast, continuous co-prescription was associated with markedly higher risks of overdose (HR 4.85, 95% CI 2.90–6.80 versus HR 2.65, 95% CI 1.45–3.80 for intermittent use) and all-cause mortality (HR 2.15, 95% CI 1.80–2.60 versus HR 1.70, 95% CI 1.35–2.15 for intermittent use) (Table S1, Fig. 1).

To further assess whether these differences reflected incremental risk attributable to concurrent benzodiazepine use among opioid-treated patients, parallel models were estimated using opioid-only exposure as the reference group. In these analyses, intermittent co-prescription remained associated with higher hazards of falls and fractures (HR 1.45, 95% CI 1.08–1.95) and hospitalizations (HR 1.15, 95% CI 1.02–1.30) compared with intermittent opioid-only use, whereas continuous co-prescription was associated with a higher hazard of overdose (HR 1.95, 95% CI 1.55–2.45) and all-cause mortality (HR 1.35, 95% CI 1.05–1.75) relative to continuous opioid-only exposure. Incremental associations for falls and fractures (HR 1.22, 95% CI 0.85–1.75) and hospitalizations (HR 1.10, 95% CI 0.88–1.38) did not reach statistical significance among patients with continuous use (Table S2, Fig. 1).

### Dose-Stratified Risks

**Fig. 2:**
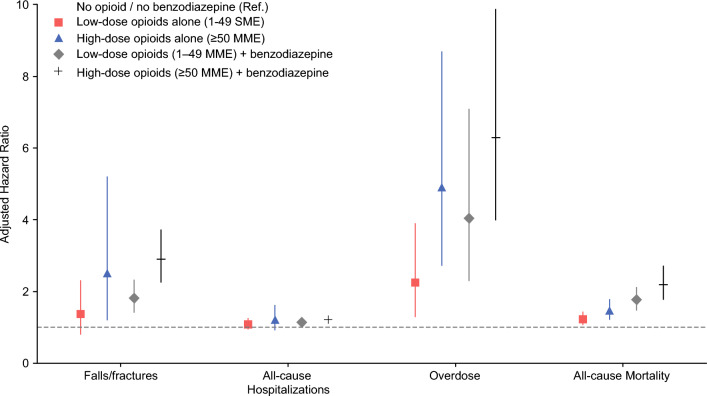
Adjusted risks of adverse outcomes by opioid dose and benzodiazepine co-prescription; adjusted hazard ratios (HRs) with 95% confidence intervals for falls/fractures, all-cause hospitalizations, overdose, and all-cause mortality among older adults with gastrointestinal cancers across five mutually exclusive exposure categories: no opioid/no benzodiazepine (reference), low-dose opioids alone (1–49 MME), high-dose opioids alone (≥ 50 MME), low-dose opioids (1–49 MME) plus benzodiazepine, and high-dose opioids (≥ 50 MME) plus benzodiazepine; models adjusted for demographics, tumor characteristics, comorbidities, mental health, cancer-directed treatment, frailty and county-level factors

In adjusted analyses stratified by opioid dose (Table S3), risks varied with dose intensity relative to no exposure. Overdose risk was observed among patients receiving high-dose opioids with benzodiazepines (HR 6.30, 95% CI 4.00–9.90), high-dose opioids alone (HR 4.90, 95% CI 2.75–8.70), and low-dose opioids alone (HR 2.25, 95% CI 1.30–3.90). Falls and fractures demonstrated elevated hazards with high-dose co-prescription (HR 2.90, 95% CI 2.25–3.74), high-dose opioids alone (HR 2.52, 95% CI 1.22–5.20), and low-dose opioids alone (HR 1.38, 95% CI 0.82–2.32). For mortality, increased risk was observed in the high-dose co-prescribed group (HR 2.20, 95% CI 1.78–2.72), as well as among patients receiving high-dose opioids alone (HR 1.48, 95% CI 1.22–1.80) and low-dose co-prescription (HR 1.78, 95% CI 1.48–2.14). Hospitalizations were modestly increased with both high-dose (HR 1.20, 95% CI 1.06–1.36) and low-dose (HR 1.15, 95% CI 1.04–1.27) co-prescription, whereas no increase was observed with high-dose opioids alone (HR 1.23, 95% CI 0.93–1.63) (Table S3, Fig. 2).

## Discussion

Older adults with GI cancers frequently experience substantial symptom burdens, including severe pain, anxiety, and insomnia, often necessitating treatment with opioid analgesics and benzodiazepines.^20^ In practice, these medications are commonly co-prescribed to manage concurrent symptoms, despite well-documented safety concerns in geriatric populations.^21^ Benzodiazepines in particular are listed on the Beers Criteria for potentially inappropriate medications in older adults due to risks of cognitive impairment, delirium, and falls, risks that are exacerbated when combined with opioids.^22^ In this national cohort study, we examined the impact of opioid–benzodiazepine co-prescribing on acute clinical outcomes among older patients with GI cancer. Two key findings emerged. First, opioid exposure was the primary driver of adverse outcomes, with both opioid-only and co-prescribed regimens associated with elevated risks relative to no exposure, and benzodiazepine co-use conferring modest incremental risk limited to hospitalization and all-cause mortality. Second, the outcome profiles differed meaningfully between intermittent versus continuous co-prescription patterns, with intermittent (short-term or episodic) overlap conferring distinct risk peaks compared with continuous long-term co-use. Together, these findings underscore both combined sedative burden between opioids and benzodiazepines and the importance of prescribing patterns in modulating patient outcomes.

While opioid exposure appeared to be the primary driver of adverse outcomes, the incremental association observed for all-cause hospitalization and mortality with opioid–benzodiazepine co-prescription likely reflected broader downstream pathways beyond direct pharmacologic toxicity. Opioids alone exert potent effects on respiratory drive, sedation, and cognition, providing a clear mechanistic basis for overdose and fall-related injury. The findings suggest that adding benzodiazepines did not meaningfully amplify these immediate risks once opioid exposure was established.^10^ In contrast, concurrent opioid–benzodiazepine use may compound longer-term vulnerability through cumulative sedation-related effects, including delirium, impaired airway protection with aspiration, functional decline, and deconditioning, each of which can independently precipitate hospitalization and increase mortality risk among older adults.^23^ Supporting this interpretation, a large study of U.S. Medicare beneficiaries demonstrated that co-administration of opioids with benzodiazepines or Z-drugs was associated with higher 90-day all-cause mortality even after excluding overdose deaths, indicating that excess risk persists through non-overdose pathways.^24^ More broadly, polypharmacy itself has been demonstrated to function as a marker of vulnerability. In a cohort of more than 3 million older adults, exposure to five or more concurrent medications was associated with an 18% higher risk of hospitalization and a 25% higher risk of mortality.^25^ Taken together, these findings suggest that opioid–benzodiazepine co-prescription selectively amplifies downstream risks related to physiologic reserve and functional resilience, manifesting as higher hospitalization and mortality even when acute harms are primarily driven by opioid exposure.

Beyond dose intensity, our data indicate that the pattern of opioid and benzodiazepine exposure shapes how adverse outcomes manifest among older adults with GI cancers. While opioid exposure establishes the underlying risk substrate, intermittent and continuous co-prescription appear to modify downstream harms in distinct ways.^6^ Intermittent or short-term overlaps, often occurring during acute symptom flares, were associated with greater risks of falls, fractures, and hospitalizations, whereas continuous co-prescription was more strongly associated with overdose and mortality.^6,26^ These patterns are biologically plausible. Episodic addition of benzodiazepines to an opioid regimen may occur in patients without established physiologic tolerance, precipitating acute sedation, impaired balance, and delirium, thereby increasing fall-related injury and hospitalization.^27^ In contrast, continuous co-prescription may reflect cumulative sedative burden over time, contributing to progressive respiratory compromise, aspiration risk, and physiologic deconditioning, which may manifest as overdose and excess mortality rather than discrete acute events.^28^ Importantly, these associations persisted when analyses were restricted to opioid-treated patients, suggesting that benzodiazepines influence how risk is expressed rather than whether risk exists. Intermittent use is therefore not benign, nor is continuous use protective; instead, each pattern confers a distinct risk profile that likely reflects differences in tolerance, monitoring intensity, and cumulative exposure.^29^ Taken together, these findings underscore that prescribing behavior and timing of opioid–benzodiazepine overlap are clinically relevant determinants of adverse outcomes in older adults with cancer.

Our findings have important clinical and policy implications for optimizing care of older adults with cancer. They underscore the need for symptom-management strategies that emphasize opioid stewardship and apply added safeguards when benzodiazepines are layered onto opioids, given the incremental risks observed for hospitalization and mortality. In geriatric oncology, multidisciplinary care should aim to minimize opioid dose and duration when feasible and avoid opioid–benzodiazepine overlap whenever possible.^30^ Early palliative care can strengthen opioid optimization, nonpharmacologic symptom strategies, and psychosocial support, reducing reflexive benzodiazepine use for anxiety or insomnia.^31^ Geriatric assessment can identify frailty, fall risk, cognitive vulnerability, and polypharmacy, informing safer prescribing.^32^ Brief balance or cognitive screening may help target higher-risk patients for alternatives (e.g., behavioral sleep interventions, shorter-acting analgesics, or cautious adjuncts such as low-dose gabapentinoids).^33,34^ Prescribing coordination between oncology and primary care—supported by prescription-drug-monitoring programs—can improve recognition of concurrent sedative exposure.^35^ Regular medication review (ideally pharmacist- or geriatrician-supported) can facilitate dose optimization and benzodiazepine tapering/deprescribing consistent with geriatric guidance.^36^ At the system level, adherence to Beers Criteria and embedding opioid-safety/co-prescribing metrics and decision-support alerts may improve safety, aligned with CDC and Food and Drug Administration (FDA) cautions on concurrent sedatives.^37,38^

Several considerations merit attention when interpreting these findings. Use of SEER–Medicare claims limited access to certain clinical details, such as functional status and symptom burden, which may influence both prescribing and outcomes, and prescription fills may not perfectly capture actual medication use. Outcomes such as overdose or falls, identified through claims codes, may also be underreported. Although residual confounding by indication cannot be fully excluded in observational analyses, particularly for broader outcomes such as all-cause hospitalization and mortality, we adjusted for a comprehensive set of demographics, as well as tumor-related, socioeconomic, clinical, and treatment factors, to mitigate this risk. The cohort was restricted to older Medicare beneficiaries, which may limit generalizability but also represents a key strength by focusing on the population most vulnerable to medication-related harm. In addition, the small number of benzodiazepine-only users precluded separate analyses of this group. Despite these limitations, the large, nationally representative dataset, rigorous analytic methods, and consistency across outcomes support the robustness and clinical relevance of our findings.

In conclusion, this national cohort study demonstrated that opioid exposure was the principal driver of elevated risks of overdose, falls, hospitalization, and mortality among older adults with GI cancers, while concurrent benzodiazepine use conferred selective incremental risk for hospitalization and mortality. Importantly, risk profiles varied by prescribing pattern, with intermittent overlap associated with acute functional harms and continuous use linked to cumulative adverse outcomes. These findings highlight the need for nuanced prescribing strategies that address symptom control while minimizing downstream vulnerability. Incorporating geriatric assessment, early supportive care, and prescribing safeguards may reduce preventable harm while preserving comfort and functional independence in this high-risk population.

## Supplementary Information

Below is the link to the electronic supplementary material.

Supplementary file1 (DOCX 20 KB)
