# Supplementary material for: Double Jeopardy: Risks of Opioid–Benzodiazepine Co-prescription in Older Adults with Gastrointestinal Cancer
Source: Ann Surg Oncol. 2026 Feb 19;33(6):5693–702. doi: 10.1245/s10434-026-19233-9 (PMC13179157; doi:10.1245/s10434-026-19233-9)
Supplement: Supplementary file 1 — Supplementary file1 (DOCX 20 KB) [file 10434_2026_19233_MOESM1_ESM.docx]

**Table S1.** Adjusted hazard ratios for adverse events, stratified by 90-day continuous exposure

| **Outcome** | **Group** | **No opioids or benzodiazepines** | **Opioid only** | **Opioids and benzodiazepines** |
| --- | --- | --- | --- | --- |
|  |  |  | **HR (95% CI)** | **HR (95% CI)** |
| **Falls/fractures** | Continuous | Ref. | 1.25 (0.93–1.70) | 1.62 (1.28–2.05) |
|  | Intermittent | Ref. | 1.75 (1.42–2.15) | 2.70 (2.15–3.40) |
| **All cause hospitalizations** | Continuous | Ref. | 0.85 (0.67–1.08) | 0.98 (0.87–1.10) |
|  | Intermittent | Ref. | 1.08 (0.99–1.18) | 1.30 (1.18–1.44) |
| **Overdose** | Continuous | Ref. | 2.10 (1.20–3.60) | 4.85 (2.90–6.80) |
|  | Intermittent | Ref. | 1.42 (1.02–1.95) | 2.65 (1.45–3.80) |
| **All-cause mortality** | Continuous | Ref. | 1.42 (1.20–1.68) | 2.15 (1.80–2.60) |
|  | Intermittent | Ref. | 1.20 (0.98–1.48) | 1.70 (1.35–2.15) |

Continuous use defined as ≥90 consecutive days of opioid or benzodiazepine exposure. Models adjusted for age group, sex, race/ethnicity, cancer site, stage, Charlson comorbidity index, frailty and treatments. Reference group = no opioid or benzodiazepine exposure. Results shown separately for patients with continuous use and intermittent use.

**Table S2.** Adjusted hazard ratios for adverse events, stratified by 90-day continuous exposure

| **Outcome** | **Group** | **Opioid only** | **Opioids and benzodiazepines** |
| --- | --- | --- | --- |
|  |  | **HR (95% CI)** | **HR (95% CI)** |
| **Falls/fractures** | Continuous | Ref. | 1.22 (0.85–1.75) |
|  | Intermittent | Ref. | 1.45 (1.08–1.95) |
| **All cause hospitalizations** | Continuous | Ref. | 1.10 (0.88–1.38) |
|  | Intermittent | Ref. | 1.15 (1.02–1.30) |
| **Overdose** | Continuous | Ref. | 1.95 (1.55–2.45) |
|  | Intermittent | Ref. | 1.30 (1.10–1.55) |
| **All-cause mortality** | Continuous | Ref. | 1.35 (1.05–1.75) |
|  | Intermittent | Ref. | 1.30 (1.01–1.68) |

Continuous use defined as ≥90 consecutive days of opioid or benzodiazepine exposure. Models adjusted for age group, sex, race/ethnicity, cancer site, stage, Charlson comorbidity index, frailty and treatments. Reference group = opioid only exposure. Results shown separately for patients with continuous use and intermittent use.

**Table S3.** Adjusted hazard ratios for adverse events, by daily opioid dose and benzodiazepine use

| **Exposure group** | **Falls/fractures** | **All-cause hospitalizations** | **Overdose** | **All-cause mortality** |
| --- | --- | --- | --- | --- |
|  | **HR (95% CI)** | **HR (95% CI)** | **HR (95% CI)** | **HR (95% CI)** |
| **No opioid or benzodiazepine** | Ref. | Ref. | Ref. | Ref. |
| **Low-dose opioids alone (1–49 MME)** | 1.38 (0.82–2.32) | 1.09 (0.95–1.26) | 2.25 (1.30–3.90) | 1.24 (1.08–1.44) |
| **High-dose opioids alone (≥50 MME)** | 2.52 (1.22–5.20) | 1.23 (0.93–1.63) | 4.90 (2.75–8.70) | 1.48 (1.22–1.80) |
| **Low-dose opioids (1–49 MME) + benzodiazepine** | 1.82 (1.42–2.34) | 1.15 (1.04–1.27) | 4.05 (2.30–7.10) | 1.78 (1.48–2.14) |
| **High-dose opioids (≥50 MME) + benzodiazepine** | 2.90 (2.25–3.74) | 1.20 (1.06–1.36) | 6.30 (4.00–9.90) | 2.20 (1.78–2.72) |

Exposure defined by daily morphine milligram equivalents (MME) and concurrent benzodiazepine use. Models adjusted for age group, sex, race/ethnicity, cancer site, stage, Charlson comorbidity index, frailty and time-varying treatments. Reference group = no opioid or benzodiazepine exposure.
